# Clinical and Economic Outcomes of Penicillin Skin Testing as an Antimicrobial Stewardship Initiative in a Community Health System

**DOI:** 10.1093/ofid/ofz109

**Published:** 2019-02-27

**Authors:** Bruce M Jones, Nenad Avramovski, Ana Maria Concepcion, Joseph Crosby, Christopher M Bland

**Affiliations:** 1 Department of Pharmacy, St. Joseph’s/Candler Health System, Inc., Savannah, Georgia; 2 SouthCoast Health, Savannah, Georgia; 3 Department of Health Sciences and Kinesiology, Georgia Southern University, Savannah, Georgia; 4 Clinical and Administrative Pharmacy, University of Georgia College of Pharmacy, Savannah, Georgia

**Keywords:** antimicrobial stewardship, penicillin allergy

## Abstract

**Background:**

Penicillin skin testing (PST) is a novel way to reduce the use of broad-spectrum agents in penicillin-allergic patients. This study evaluated the outcomes of patients with antimicrobials prescribed with and without PST in a community health system.

**Methods:**

We performed a quasi-experimental study that compared an intervention group of 100 patients who completed PST over an open enrollment period beginning January 2016 with a matched control group of 100 patients who were penicillin allergic. Patients in the control group were matched to infection diagnosis codes of members of the PST group and randomly selected and matched on a 1:1 basis. The primary outcome was noncarbapenem beta-lactam days of therapy (DOT). The secondary outcome assessed the average cost of antimicrobial therapy for the intervention group before and after PST.

**Results:**

Seventy of the 98 patients (71%) who tested negative had changes directly made to their antimicrobial regimens. Beta-lactam DOT for the PST group were 666/1094 (60.88%, with 34.82% being a penicillin specifically). Beta-lactam DOT for the control group consisted of 386/984 (39.64%, with 6.4% being a penicillin specifically). The chi-square test of homogeneity for beta-lactam DOT between the 2 groups was significant (*P* < .00001). Changes to the antimicrobial regimen after PST saved the average patient $353.03 compared with no change in the pre-PST regimen (*P* = .045).

**Conclusions:**

PST led to immediate antimicrobial de-escalation in the majority of patients who tested negative. This led to a significant increase in beta-lactam usage, specifically penicillins. These benefits were also associated with significant cost savings to patients.

Penicillin allergy is one of the most frequently reported allergies, documented in approximately 30 million patients in the United States [1, 2]. As many as 90% of these self-reported penicillin allergies are inaccurate [3]. Incorrect penicillin allergies therefore are a major barrier to antimicrobial stewardship, with many patients being likely to receive broad-spectrum or second-line antimicrobial therapy [1, 4–6]. There are also significant clinical and economic implications, including increased antimicrobial resistance, overall cost of care, increased length of stay, and ultimately mortality [7]. Penicillins and cephalosporins are the drugs of choice for many areas of infectious diseases, including surgical prophylaxis, methicillin-susceptible *Staphylococcus aureus* (MSSA) infections, invasive streptococcal infections, and sexually transmitted infections such as syphilis.

Penicillin skin testing (PST) offers a unique opportunity as part of an antimicrobial stewardship program (ASP) to address concerns around patients with listed penicillin allergies. Use of PST has been described for >50 years, primarily in outpatient allergist-run specialty clinics [8]. Recently published antimicrobial stewardship guidelines from the Infectious Diseases Society of America (IDSA) promote allergy assessment and PST to enhance the use of firstline antimicrobials [9]. PST offers a unique stewardship opportunity to not only benefit the patient for the acute episode of infection, but also provide “downstream” effects by facilitating beta-lactam prescribing and avoiding other costlier agents with potentially more adverse effects, including *Clostridioides difficile* infection. PST therefore can simultaneously promote several core elements of antimicrobial stewardship including de-escalation of therapy and parenteral to oral conversion when patients are demonstrated to not be penicillin allergic [10]. Recent studies have demonstrated the utility of using PST as a tool to support ASP. Patients who tested negative were able to have antimicrobials optimized, and an associated cost savings was shown to justify use of the test [11–13]. In July 2016, the American Academy of Allergy, Asthma, and Immunology released a position statement that PST should be performed routinely on patients with a self-reported penicillin allergy. Based on current evidence, they concluded that PST would be associated with reduced costs of care, increased patient safety, and improved patient outcomes [14].

Although recent data are encouraging, most studies performed have been within academic medical centers with significant resources, including allergists. This study examined antimicrobials prescribed before and after PST and cost savings associated with antimicrobials prescribed before and after testing to historical controls within a community health system.

## METHODS

### Study Design

We performed a quasi-experimental study at our not-for-profit community health system comparing an intervention group of 100 adult patients who completed PST for a self-reported penicillin allergy over an open enrollment period from January 2016 to January 2017. This group was compared with a matched control group of 100 patients from 2015–2017 who had a listed penicillin allergy and infectious diseases (ID) consultation. ID consultation was specifically chosen as a criterion for the control group to help identify patients with an acute infection necessitating antimicrobials. Patients in the control group were matched to the infection diagnosis codes of the members of the intervention group and then randomly selected using a random number generator, matched on a 1:1 basis.

### Testing Procedure at Facility

St. Joseph’s/Candler Health System is a 714-bed community-based regional referral health system in Savannah, Georgia. The health system consists of 2 hospitals, Candler Hospital and St. Joseph’s Hospital, with all patients from the study being inpatients at Candler Hospital. Following a recommendation from the Antimicrobial Management Program (AMP) committee and approval by the Pharmacy and Therapeutics committee, PST was added to the formulary. We previously described our institutional protocol in the literature for a pharmacist-managed, nursing-performed model. The protocol includes specifics on performing the test, interpreting the test, and documentation of the results [12]. Our 384-bed hospital has 1 full-time Infectious Diseases pharmacist (ID PharmD) with a weekly rotating ID physician who provides AMP support.

The current process involves the ID PharmD evaluating all patients prescribed PST for exclusions for use and any medications (eg, antihistamines) that would interfere with conducting the test. Currently, ordering of PST is restricted to ID physicians and recommendations from the AMP team. A thorough patient history is performed by interviewing the patient to determine specifics on the allergy history, as well as any other beta-lactam tolerated since. Patients were excluded from testing if they had a history of anaphylaxis to beta-lactam agents within the last 10 years, anaphylaxis due to any cause within the previous 4 weeks, history of severe skin reaction associated with the use of beta-lactam agents, or a skin condition that could interfere with the accurate reading of test results. Patients who were severely immunocompromised (cystic fibrosis, neutropenic [ANC < 1000/mm^3^, HIV positive with CD4 < 500 cells/uL]) were also excluded from testing. There is controversy about patients receiving H1 antihistamines and other medications that could impact the results of the positive histamine control and how long these medications must be held before testing. Currently we recommend holding H1 antihistamines for at least 48 hours before PST. Although receipt of H2 antihistamines specifically is controversial, we have previously evaluated patients tested at our facility and found that patients who received H2 antihistamines, corticosteroids, leukotriene receptor antagonists, tricyclic antidepressants, and/or immunosuppressants before PST did not interfere with histamine reactivity, regardless of when the medications were administered before testing [15]. Therefore, we performed PST on patients receiving these medications.

**Figure 1.**
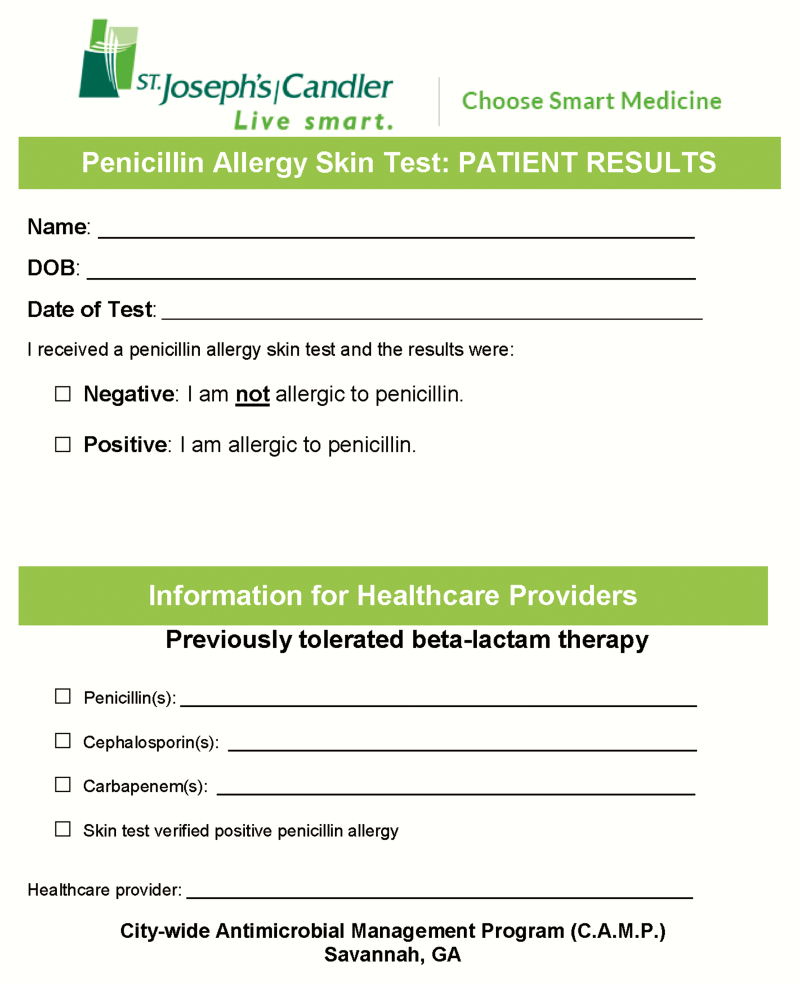
Penicillin skin test patient results wallet card.

Once a patient has been evaluated by the ID PharmD and deemed an appropriate candidate for testing, a time is scheduled with the nurse to perform the testing. The products are then profiled and prepared by the ID PharmD and taken to the bedside, where the ID PharmD assists the nurse in all aspects of the test. PST consists of a 3-step process (with the third oral amoxicillin challenge step being optional) that takes approximately 45 to 60 minutes to complete. The nurse administers each step of the procedure, and both the nurse and ID PharmD interpret each step and are trained to recognize a reaction. Once the procedure is complete, the nurse is responsible for monitoring the patient for signs or symptoms of allergic reaction, whereas the ID PharmD is responsible for calling the prescribing physician and receiving new orders for antimicrobials (if needed), updating patient allergies in the electronic medical record, and patient education. At our facility, if a patient tests negative, the allergy is removed from the electronic health record, and an uncoded place holder is added detailing the date patient was tested and that they are no longer allergic to penicillin. Patients are counseled to notify other providers (primary care providers, dentists, pharmacists) that they have been tested and, if negative, are no longer allergic to penicillin. The physician also plays an important role in counseling the patient on the importance of removing the allergy. Patients tested received a wallet card we developed detailing results of the PST (Figure 1).

### Data Collection

All patients were reviewed retrospectively with de-identified data recorded into an electronic data capture system. The following data were collected on each patient: demographics including age, sex, weight, and race. A complete allergy history was taken from the medical record to include any subjective information given by the patient that would have been available to the prescriber. Indication for antimicrobials was documented, as well as corresponding diagnostic-related group codes. For patients who had PST performed, we collected the date and location performed, specialty of the ordering provider, and result of the test. Antimicrobial therapy and duration were documented for each patient, as well as changes made to therapy in the PST group. Cost data were determined by using acquisition costs for each individual agent and multiplying based off of number of doses administered.

### Study Outcomes and Statistical Analysis

The primary outcome was beta-lactam days of therapy (DOT), defined as either a penicillin or cephalosporin (not carbapenem). This was retrospectively evaluated based on the antimicrobial regimens prescribed in both groups. The secondary outcome assessed the average cost of antimicrobial therapy before and after PST in the intervention group. Cost was determined by the acquisition cost of individual antimicrobial agents and multiplied by number of doses received for each individual agent. Each patient tested was given an additional $140 charge to account for the average drug supply cost of performing PST. To determine if there was a difference in cost of therapy, the final sum was compared with a theoretical cost if no changes in therapy had occurred.

Descriptive statistics were reported as counts/percentages for discrete patient variables and means/standard deviations for continuously measured patient variables. The research hypothesis concerning the primary outcome—overall percentage of beta-lactam days out of total antimicrobial days per group—was tested using the chi-square test of homogeneity to determine whether group and percent days of beta-lactam therapy were statistically different from each other. The research hypothesis concerning the secondary outcome—the average cost of antimicrobial therapy in the intervention group that is attributable to PST—was tested using a paired-samples *t* test. All inferential test results were evaluated for statistical significance at *P* < .05.

## RESULTS

### Baseline Characteristics

From January 2016 to January 2017, 116 patients had consults ordered for PST evaluation. Sixteen patients did not meet internal inclusion/exclusion criteria for testing and were not tested. Nine patients met internal exclusion criteria and were not candidates for testing. Six patients were able to be changed to penicillin by thorough allergy review with the patient. One patient had a negative histamine reaction on the puncture test and was unable to complete the procedure. The remaining 100 patients were all tested, with 98 testing negative. Seven of the 98 patients who tested negative had the optional oral amoxicillin challenge performed, of which all were negative. Fifty of the 100 patients tested were from direct recommendation from the ID PharmD. For the control group, there were 605 visits between 2015 through 2017 from patients with a listed penicillin allergy and ID consultation. There were 436 unique patients who were matched 1:1 to the infection diagnosis codes of the PST patients. Table 1 displays the background demographics of the 2 groups. The main difference between groups was the higher percentage of patients in the control group reporting rash (32%) compared with the PST group (8%).

**Table 1.** Patient Demographics

Demographic	PST (n = 100)	Control (n = 100)
Age, mean, y	61	65
Weight, mean, kg	87	87
Sex, No.		
Male	41	29
Female	59	71
Length of stay, median, d	8	9
Race/ethnicity, No.		
Caucasian	71	65
African American	25	29
Asian	3	0
Hispanic	0	5
Unable to obtain	1	1
Allergy history, No.		
Unknown	30	20
Swelling	14	10
Hives	23	8
Anaphylaxis (>10 y ago)	10	10
Breathing difficulty	3	5
Hives and swelling	2	0
Itching	1	4
Rash	8	32
Passed out	2	2
Other	7	9
Location PST performed, No.		
Intensive care unit	19	
Progressive care unit	16	
Medical/surgical floor	47	
Oncology floor	13	
Rehab	2	
Emergency department	1	
Labor and delivery	1	
Outpatient surgery	1	
Infection type, No.		
Skin and soft tissue	32	
Respiratory	25	
Urinary tract	14	
Cardiovascular	10	
Gastrointestinal	11	
Central nervous system	2	
Fever of unknown origin	4	
Sexually transmitted	1	
No infection	1	

Abbreviation: PST, penicillin skin testing.

### Primary and Secondary Outcomes

Seventy out of the 98 patients who tested negative for PST (71%) had changes directly made to their antimicrobial regimens immediately after PST. The most common change was from carbapenems to penicillins (34/70). Other changes to a beta-lactam after a negative test included change from a higher-generation cephalosporin to a lower-generation cephalosporin (13/70), fluoroquinolone (10/70), vancomycin (6/70), and aztreonam (2/70). For the primary outcome, beta-lactam DOT for the PST group were 666 out of 1094 (60.88%, with 34.82% being a penicillin specifically). Beta-lactam DOT for the control group consisted of 386 out of 984 (39.64%, with 6.4% being a penicillin specifically). The chi-square test of homogeneity for beta-lactam DOT between the 2 groups was significant (*P* < .00001), demonstrating a greater number of beta-lactams utilized in PST group. For the secondary outcome of average cost of antimicrobial therapy before and after PST in the intervention group, changes to the antimicrobial regimen after PST saved the average patient $353.03 compared with no change in pre-PST regimen (*P* =.045), including patients who received no changes to their antimicrobial regimen. Specifically, for the patients who received antimicrobial changes after PST (n = 70), the cost savings increased to an average of $556.91 per patient.

## Discussion

Antimicrobial allergies represent a significant obstacle in the management of infectious diseases. Coupled with antimicrobial resistance, penicillin allergies limit agent choices, resulting in increased morbidity and mortality. Studies have demonstrated the vast majority of self-reported allergies to be incorrect upon in-depth assessment or formal testing [3]. Concerted efforts by practitioners to reconcile these allergies to provide maximal benefit for the patient’s infectious disease and to prevent adverse effects of non-beta-lactam agents are crucial.

The 2016 IDSA ASP Implementation Guideline provides a weak recommendation that allergy assessment should be promoted by an ASP when appropriate, based on low-quality evidence. Recent publications documenting the benefit of penicillin allergy assessment and skin testing overall strengthen the data on using penicillin allergy assessment, and skin testing specifically, as a primary stewardship intervention. However, most of these studies documented benefit in academic medical centers with the use of a trained allergist or dedicated allergy service, which does not represent most centers providing care in the United States.

Our data demonstrate both a clinical and economic benefit to PST within a community health system without allergy services. Approximately 3 out of 4 patients in the PST group received beta-lactam therapy after negative testing, which is consistent with other published studies [12, 14]. Change specifically to a penicillin occurred in 34.8% of PST patients vs 6.4% of control patients. A significant benefit was also demonstrated by the BLAST investigators, where 32% of PST patients received penicillin in the intervention period after initial training of pharmacists by allergists vs 11% in the nonintervention period [11]. This is significant as our center more closely represents most hospitals nationally, potentially allowing translation of benefit to resource-restricted centers wishing to initiate PST services.

Nearly 100% of all patients receiving PST had a negative result. This is consistent with other studies recently published evaluating PST services [11–13]. Additionally, the histamine nonreactivity rate was extremely low in our study, allowing for maximal benefit of PST. Higher histamine control nonreactive rates have been published in the literature with risk factors such as intensive care unit stay, corticosteroid use, or histamine-2 receptor antagonist use [13]. More research is required to further delineate risk factors for negative histamine controls to limit time and resources.

An important component of PST, especially within inpatient settings with ASPs, is the real-time antimicrobial changes associated with negative testing through active stewardship intervention. Retrospective analysis of our local data demonstrated that documented penicillin allergy was responsible for 40% of empiric carbapenem prescribing, providing an opportunity for allergy assessment and PST to serve as a viable carbapenem-sparing strategy. Nearly 75% of patients were changed to a beta-lactam immediately upon negative PST, primarily through intervention of the ID PharmD pharmacist working directly with the team caring for the PST patient. The most common change in our facility was de-escalation from carbapenems to piperacillin-tazobactam. Aztreonam has been another target of stewardship programs for allergy assessment and skin testing due to its high overall cost and typically lower overall susceptibility rates in *Pseudomonas* species [16]. Heil et al. demonstrated similar findings within an ID fellow service, where 84% of patients upon negative PST received antimicrobial changes. A major advantage of providing PST services within an ASP or ID consult team is the real-time stewardship benefit of de-escalation to beta-lactams, which often are a narrower therapy or a drug therapy of choice for certain patients (ie, MSSA endocarditis).

Currently, there are minimal data regarding PST cost-effectiveness. King et al. demonstrated a ~$300/patient cost savings in patients changed to a beta-lactam in hospitalized patients receiving PST, which is similar to our findings [17]. Our data demonstrated an average cost savings of approximately $350 per patient tested based on medication costs, including the cost of the test. These savings are associated with every patient tested, including patients who are positive and patients who do not have changes to their antimicrobial regimen. In patients who directly had changes, the cost savings increased to $556.91 per patient. Some patients could not be changed due to culture results, demonstrating the need for a non-beta-lactam agent (ie, carbapenem for bacteremia with an extended-spectrum beta-lactamase-producing strain of *Enterobacteraciae*). These are likely conservative cost savings estimates considering that the true cost of using non-beta-lactam agents has been well documented with regards to morbidity and mortality due to decreased effectiveness or increased adverse effects. With ASPs now being required by regulatory agencies, smaller resource-limited programs are forced to decide which services offer the most impact for their patient population. PST may “compete” with other beneficial ASP initiatives, such as rapid diagnostic testing, for implementation. One primary advantage of PST is that although most ASP interventions result in a 1-time benefit for the acute infectious episode of the patient, penicillin allergy de-labeling will result in a longitudinal benefit in most cases. The deleterious ramifications of possessing a penicillin allergy are well described [4, 18]. De-labeling the allergy would benefit the patient postdischarge, when patients are readmitted requiring antimicrobial therapyin outpatient settings when experiencing other episodes of infectious disease or when requiring antimicrobial prophylaxisfor dental or surgical procedures. More data are needed on the long-term pharmaco-economic outcomes of PST.

Although programs with allergy services are optimal, most allergists practice in an outpatient setting with limited numbers available in most health systems for inpatient PST [19]. Successful models utilizing allergists, ID fellows, pharmacists, and nurses have been published, primarily within academic medical centers [11, 13]. Our data are reassuring, demonstrating PST as a beneficial clinical and economic service within a community health system without allergy availability for PST. It is important to note that PST is part of a broader initiative including penicillin allergy assessment. Upon allergy assessment, many patients will not require PST and can safely be administered a beta-lactam in cases where the allergy history is incorrect or given a graded challenge if the patient is at low risk overall for reaction. PST remains the best option for patients with confirmed IgE-mediated reaction to penicillins and should be offered to inpatients for de-labeling if possible, allowing for beta-lactam prescribing after discharge. Each center must assess their available resources and develop a process for implementing penicillin allergy assessment and skin testing. Additionally, programs involving pharmacists should evaluate state laws to determine appropriate roles within the PST process, especially regarding administration of the PST components [20].

There are a few limitations to our study. First, due to the observational nature, patient selection bias could not be controlled for in the PST patients. The control group was required to have ID consultation for this reason to help ensure that patients were acutely infected. This would also account for the higher percentage of patients in the control group having “rash” as the listed reaction to penicillin. Second, our services were performed at a single center with an established PST program. Third, our cost savings applied directly to antimicrobial use during acute admission and did not take into account personnel time and overhead. The demonstrated cost savings, however, did not take into account antimicrobial use during future admissions, which is likely to make PST more cost-effective. Additionally, our cost savings were based on local contract prices, and therefore assessment by each facility will be required to ascertain cost savings.

## Conclusions

In conclusion, PST led to immediate antimicrobial de-escalation in the majority of patients who tested negative within a community health system, leading to a significant increase in beta-lactam usage, specifically penicillins. These benefits were also associated with significant antimicrobial cost savings to patients, justifying the cost of performing PST. Further study is required to evaluate overall clinical and economic benefit in community health systems with limited resources.
